# Aqueous fish extract increases survival in the mouse model of cytostatic toxicity

**DOI:** 10.1186/1756-9966-27-81

**Published:** 2008-12-04

**Authors:** Elmir Omerovic, Malin Linbom, Truls Råmunddal, Ann Lindgård, Ingrid Undeland, Ann-Sofie Sandberg, Bassam Soussi

**Affiliations:** 1 Wallenberg Laboratory at Sahlgrenska Academy, Göteborg University Göteborg, Sweden; 2 Department of Chemical and Biological Engineering, Food Science, Chalmers University of Technology, Göteborg, Sweden; 3 UNESCO Chair, CAMS, Sultan Qaboos University, Muscat, Oman

## Abstract

**Background:**

Treatment of cancer patients with anthracycline antibiotic doxorubicin (DOX) may be complicated by development of acute and chronic congestive heart failure (CHF), malignant arrhythmias and death. The aim of this study was to test whether an aqueous low molecular weight (LMW) extract from cod muscle decreases acute mortality in the mouse model of acute CHF caused by DOX.

**Methods:**

A LMW fraction (<500 Da) of the aqueous phase of cod light muscle (AOX) was used for treatment of male BALB/c mice (~25 g, n = 70). The animals were divided into four groups, DOX + AOX (n = 20), DOX + saline (NaCl) (n = 30), NaCl + AOX (n = 10) and NaCl only (n = 10). Echocardiography was performed in the separate subgroups (DOX treated n = 6 and controls n = 6) to verify the presence and the grade of acute CHF. The cod extract was delivered by subcutaneously implanted osmotic minipumps over the period of 2 weeks. High-dose injection of DOX was administered to randomly selected animals. The animals received single intraperitoneal injection of DOX (25 mg/kg) and were followed over two weeks for mortality.

**Results:**

Mortality rate was 68% lower (p < 0.05) in the mice treated with the extract. The analyses of cod extract have shown strong antioxidative effect *in vitro*.

**Conclusion:**

The aqueous LMW cod muscles extract decreases mortality in the mouse model of DOX induced acute CHF. This effect may be mediated by cardioprotection through antioxidative mechanisms.

## Introduction

The anthracycline antibiotic doxorubicin (DOX) is one of the most effective antitumor agents against human malignancies such as leukemia, lymphomas and many solid tumors [1-3]. However, the treatment of cancer patients with DOX may be complicated by deleterious side effects. One of the most feared side effects of DOX is a direct damage to the heart which may lead to the development of acute and chronic congestive heart failure (CHF), malignant arrhythmias and death [4,5]. The acute form of cardiotoxicity may occur within a week of the treatment and is not dose-depended, indeed, it may occur after a single dose of the agent. On the other hand, the incidence of the chronic CHF at specific cumulative doses of doxorubicin include 0.4% at 400 mg/m^2 ^of body surface area, 7% at 550 mg/m^2^, and 18% percent at 700 mg/m^2 ^[3]. Electrophysiologic abnormalities are frequent and are detected as ECG changes in 20–30% of the patients in form of non-specific ST and T wave changes, T wave flattening, decreased QRS voltage and/or prolongation of the QT interval. Arrhythmias, including ventricular and supraventricular tachycardias are seen in 0.5–3% of patients with overall incidence of 0.7% [2]. Many different cardioprotective agents have been tested for prevention of DOX toxicity experimentally and clinically but with limited success[6]. Considering the essential role of iron and the doxorubicin-iron complex, iron chelators have been developed against cardiotoxicity where dexrazoxane was found to be the most promising drug[7]. Myocardial damage is a consequence of direct DOX interference with important intracellular homeostatic processes primarily mediated by increased intracellular oxidative stress[8]. Intrinsic antioxidative systems in the cells are normally able to reduce the damage caused by oxidative stress. Compared to other organs, however, the heart has inherently lower potential to protect itself from the free radicals due to the limited number of anti-oxidative systems in the cardiomyocytes[9]. It is therefore hypothesized that treatment with antioxidants may play an important role in preventing myocardial damage. The mouse model of acute DOX toxicity is a simple and readily available small animal model particularly suitable for screening studies for different cardioprotective interventions[10,11]. In the recent years, there has been an increased research interest to develop cardioprotective and other pharmacological therapies from natural sources like animals and plants of marine origin. A well known example of such a promising clinical application in the field of cardiovascular medicine are the fish-derived long chain n-3 polyunsaturated fatty acids[12,13]. There is evidence that also aqueous fish-derived substances, such as taurine, have cardioprotective effects[14]. It has been speculated that these observations are caused by a radical scavenging mechanism[15]. We hypothesize that aqueous fish muscle extract may improve survival in mouse model of acute heart failure.

## Methods

### Animals

The Animal Ethics Committee of the University of Göteborg approved the animal experiments. Male BALB/c mice (B&K Universal AB) weighing ~25 g were used in all experiments. Fresh whole cod (Gadus morhua) was obtained from Leröy Allt i Fisk (Göteborg, Sweden).

### Preparation of extract from cod muscle

The cod was manually filleted and the light muscle was separated from skin and dark muscle. The light muscle was minced, packed in 200 ml polypropylene centrifuge bottles (200 g in each) and centrifuged at 15 000*g for 2 h at 4°C. The supernatant (press juice) was filtered through a filter paper (1010, Munktell's, Swedish Filter paper, Grycksbo pappersbruk AB, Sweden) and frozen immediately at -80°C. Using a stirred 400 ml Amicon ultra filtration cell (Model 52, Amicon Corporation, Danvers, MA, USA), 50 ml of thawed cod muscle press juice was filtered through a 500 Da ultrafiltration membrane (Millipore Corporation, Bedford, MA, USA) at 50 psi and 4°C. The first 25 ml of filtrate were collected and the pH was adjusted to 7.2 with 1 M NaOH. The filtrates were then immediately frozen at -80°C.

### Animal model

To evaluate whether the cod extract may be delivered subcutaneously without provocation of an inflammatory response, three animals were treated with the extract delivered by subcutaneously implanted osmotic pumps (Alzet, Durect Corp., Cupertino, USA, Model NO. 2002). The animals were followed up for 10 days and were inspected for signs of skin irritation and possible abscess formation. No signs of inflammation were found in the region after 10 days of follow-up.

### Transthoracal echocardiography

One day after injection of DOX, echocardiography was performed in the subgroup of DOX treated mice (n = 6) and compared to the normal animals (n = 6). The purpose of this examination was to validate the model of acute DOX-induced cardiotoxicity i.e. to detect and measure the extent of cardiac damage. The examination was performed according to the previously described method[16]. Echocardiographic images were obtained using a commercially available ultrasound system (ATL, Philip Medical System, Best) equipped with a 15 MHz linear transducer.

### Survival study

**Figure 1:**
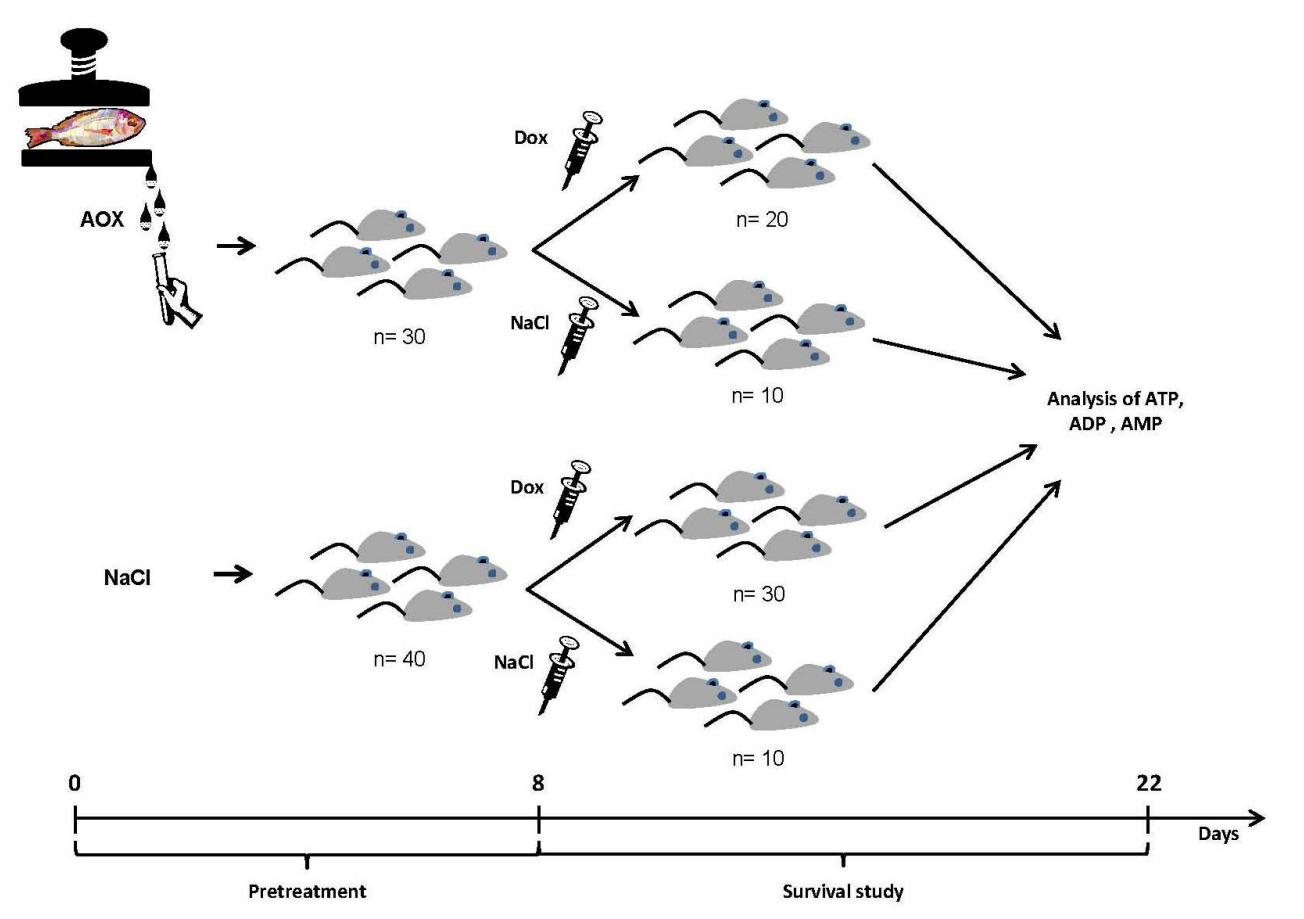
Schematically depicted experimental study design. The press-juice consisting of LMW aqueous extract of the codfish was prepared and administered to the randomly selected mice eight days prior to the administration of single high-dose injection of doxorubicin. The animals were followed-up during two weeks for survival. ATP = adenosine-tri-phosphate, ADP = adenosine-diphosphate, AMP = adenosine-monophosphate, AOX = codfish extract, DOX = doxorubicin, NaCl = saline

A total of 70 mice were divided into the following four groups: mice treated with DOX and cod muscle extract (DOX + AOX, n = 20), mice treated with DOX receiving saline (DOX + NaCl, n = 30), normal mice treated with cod extract and saline (AOX + NaCl, n = 10) and normal mice treated only with saline (NaCl, n = 10) (Figure 1). The average daily doses of cod extract and saline given to the mice were 12 μL/day. The AOX + DOX and the AOX + NaCl groups were pre-treated with AOX by means of osmotic pumps. The DOX + NaCl and NaCl groups received 0.9% solution of NaCl in the same manner. After 8 days of pretreatment, the DOX + AOX and the DOX + NaCl group received a single dose of DOX (25 mg/kg, Pfizer, Sollentuna, Sweden) by means of intraperitoneal injection. The AOX + NaCl and NaCl groups were injected with 0.9% solution of NaCl in the same manner. Mortality and health conditions were monitored daily for 14 days.

### Analysis of ATP, ADP and AMP

The concentrations of myocardial ATP, ADP, and AMP were determined by high performance liquid chromatography (HPLC) from the animals that have survived 2 weeks according to the method previously described[17]. The heart was explanted and immediately frozen in liquid nitrogen. The samples were maintained in a -134°C until final analysis. After preparation of the samples, the metabolites were separated after polarity in a column (Phenomenex^® ^Luna 5 u C18 colonn) and analyzed on standard HPLC system (Gynkotek, Germering Germany).

### Analysis of moisture content, pH and conductivity

The moisture content of the cod extract was measured using a HA300 Moisture Balance (Precisa balance 310 M, Zurich, Switzerland) (n = 2). The moisture results were expressed as percentage of wet weight. A Hamilton double pour electrode (Hamilton Double Pore, Bonaduz, Switzerland) in conjunction with a pH-meter (Radiometer analytical PHM210, Villeurbanne Cedex, France) was used for pH measurements. Conductivity was measured with a conductivity meter (CDM 210, Meter Lab, Radiometer Analy SAS, Villeurbanne Cedex, France).

### Analysis of protein content

Total protein measurements of the cod extract were done according to Lowry et al[18] (n = 2). Protein content was expressed as mg/mL of sample.

### Analysis of total lipids

The method described by Lee et al[19] was used to determine the total lipids in the cod extract using chloroform and methanol (1:1) as the extraction solvent (n = 2).

### Analysis of ascorbic acid and uric acid

Ascorbic acid and uric acid of the cod extract was analyzed with HPLC using an electrochemical detector[20], with modifications according to Gunnarsson et al. [21] (n = 3). Both ascorbic acid and uric acid results are expressed as μM.

### Analysis of amino acids

Total amino acids, free amino acids and some di-peptides like carnosine and anserine in the cod extract were analyzed with HPLC according to Fontaine et al. [22] (n = 2). Results were expressed as g/kg.

### Analysis of the capacity of the cod extract to scavenge peroxyl radicals and to prevent ROS formation from human monocytes

The antioxidative capacity of the cod extract was analyzed quantitatively *in vitro*. These tests were based on the measurement of capacity to scavenge peroxyl radical activity using the Oxygen Radical Absorbance Capacity (ORAC) test and to scavenge reactive oxygen species (ROS) produced by phorbol myristate acetate (PMA) in stimulated human monocytes. The ORAC test, the method for isolating monocytes from human blood, and the method for analyzing whether the cod extract could prevent phorbol myristate acetate (PMA) initiated ROS formation of the monocytes is described by Gunnarsson et al[21]. In brief, the ROS-preventing effect of the cod extract was analyzed as a relative reduction in the isoluminol-enhanced chemiluminescence (CL) signal given by the monocyte-derived ROS. Thus, the CL-signal given by the monocytes in the presence of the cod extract was compared with a control assay where no extract was added.

### Statistics

Computer software (StatView 5.0.1) was used to perform standard statistical procedures. Mortality rate was tested using a 2 × 2 contingency table. Fisher's PLSD (Protected Least Significant Difference) test proceeded by one-way analysis of variance (ANOVA) was applied to detect significant differences between different treatments for interactions defined in advance. The value p < 0.05 was considered as statistically significant. All data are presented as mean ± SEM.

## Results

### Animals

All animals treated with DOX have shown lower BW (data not shown), appeared weak and lethargic. At necropsy, the most prominent gross pathologic change in the mice treated with DOX was excessive amounts of pericardial, pleural and peritoneal fluid indicating the presence of systemic as well as local toxic effect. On a gross pathological cardiac examination the hearts of the DOX treated animals were enlarged and there were signs of pericarditis with multiple adhesions of connective tissue formed between the pericardium and the inner thoracic wall. Furthermore, the epicardial surface was rich in areas of local fibrosis. These signs were not present in the control animals. No difference in the BW was found between the DOX + AOX and the DOX + NaCl groups. The DOX + AOX mice had less pronounced accumulation of pericardial, pleural and peritoneal fluid compared to the DOX + NaCl mice.

### Analyses of myocardial purine nucleotides

There was no difference between the groups concerning the amount of myocardial ATP, ADP or AMP (Table 1).

**Table 1:** Myocardial content of purine nucleotides determined by HPLC

	ATP μmol/g	ADP μmol/g	AMP μmol/g
DOX + AOX (n = 8)	16.2 ± 0.6	9.2 ± 0.8	2.7 ± 0.5
DOX + NaCl (n = 4)	18.6 ± 1.3	9.0 ± 0.3	2.5 ± 0.5
AOX + NaCl (n = 10)	16.2 ± 0.5	10.0 ± 0.4	2.8 ± 0.3
NaCl (n = 10)	15.3 ± 0.5	10.0 ± 0.3	3.0 ± 0.2

ATP = adenosine-tri-phosphate, ADP = adenosine-diphosphate, AMP = adenosine-monophosphate, AOX = codfish extract, DOX = doxorubicin, NaCl = saline

### Echocardiography

**Figure 2:**
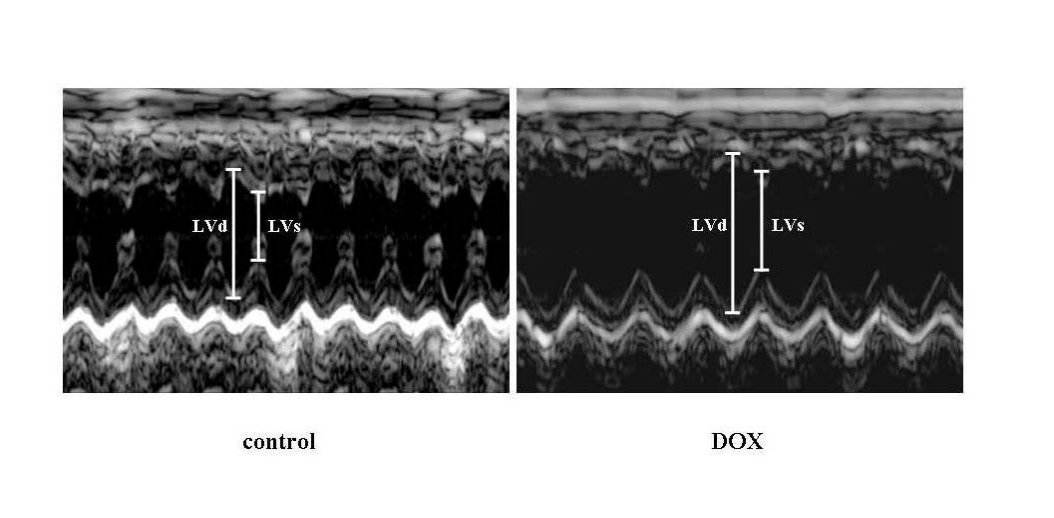
M-mode tracings of left ventricle (LV) from the mouse treated with doxorubicin (DOX) and the control mouse one day after DOX administration. LV dilatation and decreased systolic function are evident in the DOX treated mouse. LVd = left ventricular diameter in diastole, LVs = left ventricular diameter in systole

Echocardiography performed in the subgroup of the DOX-treated animals has demonstrated the presence of severely impaired LV function (Table 2). Compared to the normal controls, the DOX treated mice had lower fractional shortening (FS), cardiac output (CO), stroke volume (SV) and heart rate (HR) (all p < 0.05). There were signs of early LV remodeling with increased LV volumes in systole and diastole (both p < 0.05) in the DOX treated mice (Figure 2). These findings demonstrate the presence of severe myocardial damage induced by DOX.

**Table 2:** Echocardiography

	LVd (mm)	LVs (mm)	FS (%)	CO (ml/min)	HR (beats/min)
DOX (n = 6)	4.5 ± 0.2*	3.2 ± 0.2*	28 ± 2*	2.3 ± 1.4*	322 ± 21*
Control (n = 6)	3.7 ± 0.04	2.1 ± 0.07	43 ± 2	14 ± 1.6	406 ± 22

* p < 0.05 v. control

LVd = left ventricular diameter in diastole, LVs = left ventricular diameter in systole, FS = fractional shortening, CO = cardiac output, HR = heart rate, DOX = doxorubicin

### Survival study

**Figure 3:**
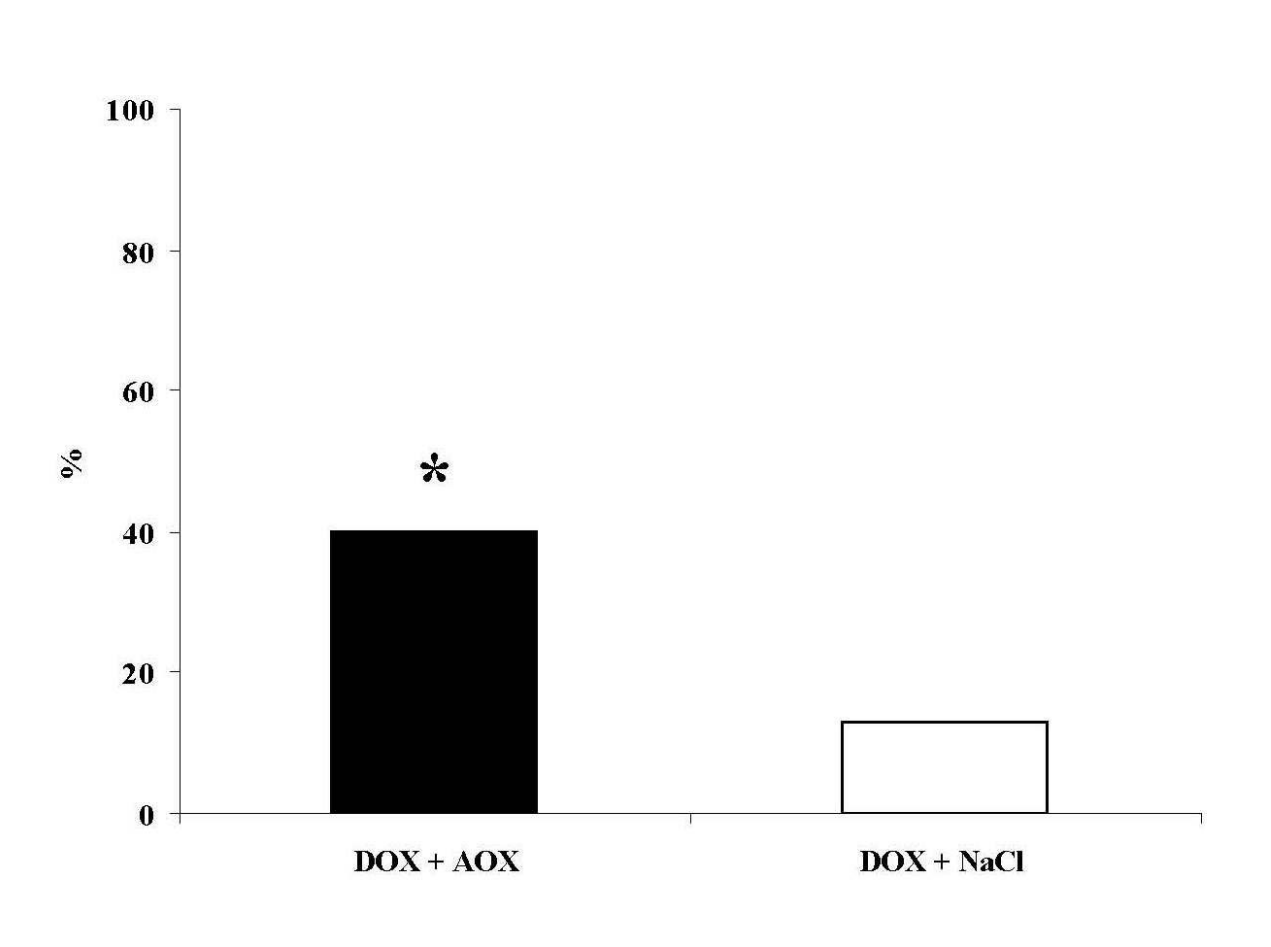
Effect of the codfish extract on acute mortality rate in the mice with doxorubicin induced acute heart failure. The treatment decreased the mortality rate by 50% compared to the control group. * p < 0.05 v. NaCl + DOX

In the DOX + AOX group 8/20 (40%) of animals were alive at 14 days after the injection of DOX compared to only 4/30 (13%) in the DOX + NaCl (p < 0.05; Figure 3). There were no deaths in the control groups that did not receive DOX.

### Compositional analyses of the cod extract

According to Table 3, the dry matter of the cod extract was very low, reflecting the LMW-character of its composition. The natural pH of the extract, prior to adjusting it to 7.2 for administration into the mice, was 6.48. The extract conductivity was 7.83 mS/cm. The extract was totally deficient of lipids, and had very low protein content (0.33 mg/mL). Since the extract was ultrafiltered it only contained small peptides (<500 Da). Two important LMW muscle antioxidants were measured, ascorbic acid and uric acid. Their content was 4.3 and 0.34 μM, respectively. Both total amino acids (Table 4) and free amino acids, including certain dipeptides (Table 5), were analyzed. As the detection limit of the total amino acid method was higher than that for the free amino acid method, much fewer amino acids are listed in Table 4 than in Table 5. Among total amino acids, only taurine, glycine, alanine and β-alanine were above the detection limit (0.5, 0.5, 0.3 and 0.2 g/L, respectively). Regarding free amino acids, the highest amount (0.55 g/L) was made up by taurine, followed by glycine (0.325 g/L), alanine (0.295 g/L), β-alanine (0.175 g/L), methylhistidine (0.095 g/L) and anserine (0.9 g/L); all above or close to 0.1 g/kg. A few minor amino acids were also detected like threonine, glutamic acid, proline, valine, methionine, and leucine.

**Table 3:** Compositional data and ORAC data of the LMW (< 500 Da) fraction of cod press juice.

Measurements	Cod extract (<500 Da)
Dry Matter % (n = 2)	1.94
Native pH (n = 2)	6.48
Conductivity (mS/cm) (n = 2)	7.83
Total lipids % (n = 2)	Not detected
Total protein (mg/mL) (n = 2)	0.33
Ascorbic acid (μM) (n = 3)	4.30 ± 0.54
Uric acid (μM) (n = 3)	0.34 ± 0.004
ORAC (mmol Trolox equivalents/l sample) (n = 8)	1.17 ± 0.06

### Antioxidant testing of the cod extract using ORAC

**Figure 4:**
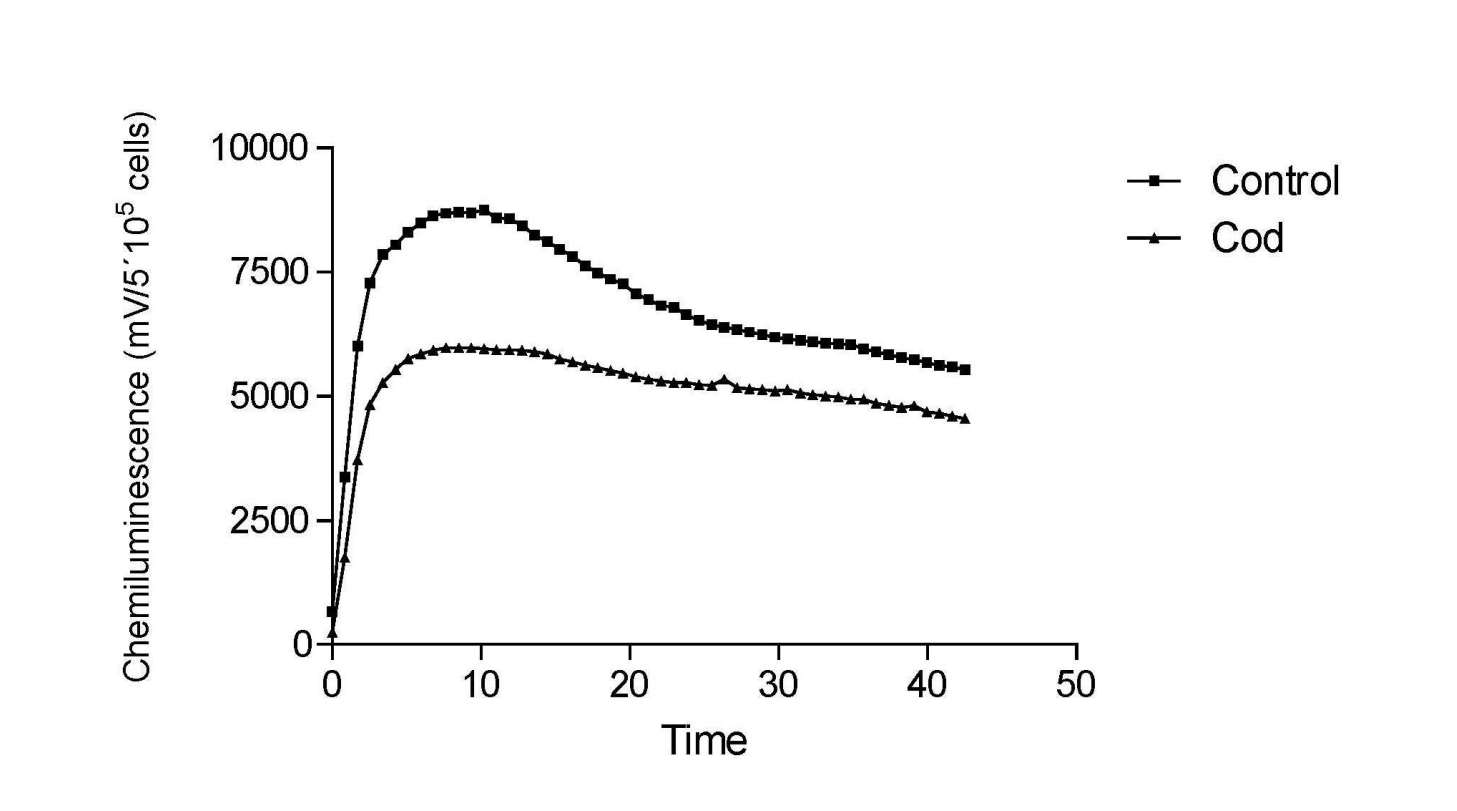
Effect of the LMW cod extract (1/100 dilution) on the chemiluminescence from monocytes induced by 10 nM of PMA. Control contains buffer instead of cod extract.

According to Table 3, the ORAC-value of the cod extract was 1.17 mmol trolox equivalents/L sample. In Figure 4, it is shown that the cod extract, at a 100-fold dilution, reduced the maximum CL-signal given by monocytes stimulated with 10 nM of PMA by about 50%.

## Discussion

The main results of the study could be summarized as follows: The LMW cod muscle aqueous extract decreased the mortality rate in mice following DOX-induced acute CHF. The extract is characterized by pronounced free-radical scavenging effects *in *vitro.

To the best of our knowledge, this is the first study to demonstrate the beneficial effect of an aqueous fish extract on survival rate in an *in vivo *animal model of acute heart failure. Cardioprotection has been in the research focus for many years. Different pharmacological and non-pharmacological strategies have been proposed to decrease myocardial damage e.g. during ischemia-reperfusion injury and cardiotoxicity. However, only few experimental interventions have been translated into a clinical practice and with a limited success. Several studies have shown beneficial cardiovascular effects of fish and fish derived substances. By far the best known are the long chain n-3 polyunsaturated fatty acids[12,13]. These fatty acids may enter the cellular membrane and alter membrane functions resulting in, e.g., antiarrhythmic effects[12]. Recent evidence indicates that dietary supplementation with fish oil preserves normal vasomotion of atherosclerotic coronary arteries and reduces damage to the myocardium after ischemia and reperfusion in animal models[23,24]. Much less is known whether aqueous components of fish, e.g. proteins, peptides, amino acids and other organic acids may protect the heart. On a wet weight basis, the aqueous phase usually contribute to 95–99.5% of the total edible parts of seafoods. Indeed, the results of the present study provide the evidence that also aqueous fish-derived compounds may play an important role in cardioprotection.

The mouse model of DOX induced acute heart failure is suitable for screening studies and is well characterized in the literature[25]. We have verified the presence of acute CHF in the animals treated with DOX demonstrating that already one day after the exposure to the high-dose DOX, the indices of LV function were severely impaired with development of early pathologic LV remodeling (Figure 2). It is therefore not surprising that the mortality rate after high-dose DOX reached ~90% in the untreated animals. Although several organ systems sustain damage in this model, the central role in the progression of the multiorgan failure is the failing heart. Acute myocardial injury is a result of pathophysiological abnormalities that are caused by direct toxic intracellular actions of DOX and include inhibition of nucleic acid and protein synthesis, release of vasoactive amines, alteration in adrenergic function, mitochondrial abnormalities, lysosomal changes, modification of sarcolemma Ca^2+ ^transport, attenuation of adenyl-cyclase, Na^+^-K^+^-ATPase, and Ca^2++^-ATPase activities, imbalance in myocardial electrolytes. However, most of the studies support the view that oxidative stress holds the central role in the development of these derangements (see ref. [8] for review). Our study does not provide the exact explanation for the possible mechanisms behind the improved survival rate. Given the fact that cardiotoxicity with acute heart failure is the primary cause of death in this model, we speculate that the treatment was primarily cardioprotective. At the cellular level, this cardioprotection may have been mediated by antioxidative effects and/or by other mechanisms. Others have shown that increasing antioxidative capacity of the heart muscle suppresses cardiotoxicity of DOX[26]. The aqueous LMW-cod extract used in this study was reported to possess strong antioxidative effects[27]. However, it has not been previously evaluated whether this extract exerts antioxidative properties under physiological conditions. The results shown in Figure 3, 4 and Table 3 provide evidence for such an effect. The data from human monocytes in Figure 4 indicate that the capacity of the cod extract to prevent ROS-formation is ~50% at a 100-fold dilution! To evaluate the contribution of some individual compounds of the cod extract to its total antioxidative capacity (ORAC value) it was compared to some previously reported ORAC values for solutions of pure ascorbic acid and uric acid at the equimolar levels to those measured in the cod extract[28]. These substances had ORAC values three orders of magnitude lower than the cod extract, indicating that other compounds in the extract appear to be involved in its radical scavenging effect and/or that antioxidative substances require other LMW-compounds present at the same time for regeneration purposes. Which are the most likely candidates in the extract that have provided organ protection and survival benefit in this study? The LMW-compounds of fish muscle that has been ascribed antioxidative properties include ascorbic acid, uric acid, glutathione, various polyamines, histidine containing dipeptides (anserine, carnosine) and free amino acids (taurine, histidine). Based on the quantitative analyses (Table 3, 4, 5) we speculate that taurine and anserine might have been responsible for the most part of the protection. Taurine is generally found in high levels in seafood[29]. It is involved in radical scavenging, membrane regulation, osmoregulation and regulation of calcium homeostasis[30]. In animal models and human trials in the settings of CHF, taurine was found to have beneficial effects on cardiac function and morphology[31,32]. Similarly, anserine has shown antioxidative effects at physiological levels in different in vitro systems[33]. We did not find any differences between the groups in regard to myocardial contents of ATP, ADP or AMP. Unexpectedly the DOX treated groups did not show lower ATP levels. One possible explanation for this result may be the selection bias since this analysis was performed only on biopsies from the surviving mice.

**Table 4:** Composition of total amino acids in Cod LMW-PJ (<500 Da).

Average g/L sample	Cod LMW-PJ (<500 Da)
Taurine	0.5 ± 0
Cysteine	<0.1
Methionine	<0.1
Aspartic acid	<0.1
Threonine	<0.1
Serine	<0.1
Glutamic acid	<0.1
Proline	<0.1
Glycine	0.5 ± 0
Alanine	0.3 ± 0
β-Alanine	0.2 ± 0
Valine	<0.1
Isoleucine	<0.1
Leucine	<0.1
Tyrosine	<0.1
Phenyl alanine	<0.1
Histidine	<0.1
Ornithine	<0.1
Lysine	<0.1
Arginine	<0.1
Hydroxyproline	<0.1

Sum	1.5

**Table 5:** Composition of free amino acids and certain dipeptides in Cod LMW-aqueous extract (<500 Da).

g/L	Cod extract (<500 Da)
Phosphoserine	<0.02
Taurine	0.55 ± 0
Phosphoethanolamine	<0.02
Urea	0.07 ± 0.01
Aspartic acid	<0.02
Threonine	0.04 ± 0
Serine	0.03 ± 0
Aspargine	<0.02
Glutamic acid	0.04 ± 0
Sarcosine	<0.02
α-Aminoadipitic acid	<0.02
Proline	0.035 ± 0.005
Glycine	0.325 ± 0.005
Alanine	0.295 ± 0.005
Citrulline	<0.02
α-Amino-n-butyric acid	<0.02
Valine	0.04 ± 0
Cysteine	<0.02
Methonine	0.02 ± 0
Cystathionine	<0.02
Isoleucine	<0.02
Leucine	0.04 ± 0
Tyrosine	<0.02
β-Alanine	0.175 ± 0.015
Phenyl alanine	<0.02
β-Aminoisobutyric acid	<0.02
L-Homocystine	<0.02
γ-Amino-n-butyric acid	<0.02
Ethanolamine	<0.02
Ammonia	0.08 ± 0
γ-Hydroxilysine	<0.02
Ornithine	<0.02
Lysine	<0.02
1-Methylhistidine	0.095 ± 0.005
Histidine	<0.02
3-Metylhistidine	<0.02
Anserine	0.09 ± 0
Carnosine	<0.02
Arginine	<0.02
Hydroxiproline	<0.02
Glutamine	<0.02

There are some limitations that deserve to be mentioned. We have not compared the efficacy of the cod muscle extract to other known cardioprotective agents such as dexrazoxane. We have not demonstrated specific cardioprotective effects of the extract in terms of cardiac function (echocardiography) or tissue structure (pathohistology). Furthermore, our study does not provide the answer to whether the survival benefit is mediated by few compounds in the extract (such as taurine and anserine) or by multiple compounds optimally mixed in this preparation.

In conclusion, the aqueous LMW cod muscles extract decreases mortality in the mouse model of DOX induced acute CHF. This effect may be mediated by cardioprotection through antioxidative mechanisms.

## Competing interests

The authors declare that they have no competing interests.

## Authors' contributions

EO participated in the study design, performed the statistical analysis and was primarily responsible for the writing of the manuscript. ML carried out animal handling and HPLC analysis and participated in the preparation of the manuscript. TR carried out echocardiographic investigations and interpretation of the data and participated in the preparation of the manuscript. AL carried out the survival study and participated in HPLC analysis. IU carried out compositional analyses and characterization of the cod extract and participated in the preparation of the manuscript. ASS carried out compositional analyses and characterization of the cod extract and participated in the preparation of the manuscript. BS conceived and initiated the study, designed the study, supervised the preparation of the manuscript and was responsible for the whole project as a senior scientist.

## References

[B1] Singal PK, Li T, Kumar D, Danelisen I, Iliskovic N (2000). Adriamycin-induced heart failure: mechanism and modulation. Mol Cell Biochem.

[B2] Frishman WH, Sung HM, Yee HC, Liu LL, Keefe D, Einzig AI, Dutcher J (1997). Cardiovascular toxicity with cancer chemotherapy. Curr Probl Cancer.

[B3] Shan K, Lincoff AM, Young JB (1996). Anthracycline-induced cardiotoxicity. Ann Intern Med.

[B4] Li T, Danelisen I, Singal PK (2002). Early changes in myocardial antioxidant enzymes in rats treated with adriamycin. Mol Cell Biochem.

[B5] Kumar D, Lou H, Singal PK (2002). Oxidative stress and apoptosis in heart dysfunction. Herz.

[B6] Dorr RT (1996). Cytoprotective agents for anthracyclines. Semin Oncol.

[B7] Swain SM, Whaley FS, Gerber MC, Ewer MS, Bianchine JR, Gams RA (1997). Delayed administration of dexrazoxane provides cardioprotection for patients with advanced breast cancer treated with doxorubicin-containing therapy. J Clin Oncol.

[B8] Singal PK, Iliskovic N, Li T, Kumar D (1997). Adriamycin cardiomyopathy: pathophysiology and prevention. Faseb J.

[B9] Singal PK, Khaper N, Palace V, Kumar D (1998). The role of oxidative stress in the genesis of heart disease. Cardiovasc Res.

[B10] Robert J (2007). Long-term and short-term models for studying anthracycline cardiotoxicity and protectors. Cardiovasc Toxicol.

[B11] Zhu W, Shou W, Payne RM, Caldwell R, Field LJ (2008). A mouse model for juvenile doxorubicin-induced cardiac dysfunction. Pediatr Res.

[B12] Grundy SM (2003). N-3 fatty acids: priority for post-myocardial infarction clinical trials. Circulation.

[B13] Leaf A, Kang JX, Xiao YF, Billman GE (2003). Clinical prevention of sudden cardiac death by n-3 polyunsaturated fatty acids and mechanism of prevention of arrhythmias by n-3 fish oils. Circulation.

[B14] Yamori Y, Liu L, Ikeda K, Miura A, Mizushima S, Miki T, Nara Y (2001). Distribution of twenty-four hour urinary taurine excretion and association with ischemic heart disease mortality in 24 populations of 16 countries: results from the WHO-CARDIAC study. Hypertens Res.

[B15] Schaffer S, Takahashi K, Azuma J (2000). Role of osmoregulation in the actions of taurine. Amino Acids.

[B16] Omerovic E, Bollano E, Andersson B, Kujacic V, Schulze W, Hjalmarson A, Waagstein F, Fu M (2000). Induction of cardiomyopathy in severe combined immunodeficiency mice by transfer of lymphocytes from patients with idiopathic dilated cardiomyopathy. Autoimmunity.

[B17] Soussi B, Lagerwall K, Idstrom JP, Schersten T (1993). Purine metabolic pathways in rat hindlimb perfusion model during ischemia and reperfusion. Am J Physiol.

[B18] Lowry OH, Rosebrough NJ, Farr AL, Randall RJ (1951). Protein measurement with the Folin phenol reagent. J Biol Chem.

[B19] Lee CM, Trevino B, Chaiyawat MA (1996). Simple and Rapid Solvent Extraction Method for Determining Total Lipids in Fish Tissue. J AOAC Int.

[B20] Margolis SA, Paule RC, Ziegler RG (1990). Ascorbic and dehydroascorbic acids measured in plasma preserved with dith iothreitol or metaphosphoric acid. Clin Chem.

[B21] Gunnarsson G, Undeland I, Sannaveerappa T, A-S S, Lindgård A, Mattsson-Hultén L, Soussi B (2006). Inhibitory effect of known antioxidants and of press juice from herring (Clupea harengus) light muscle on the generation of free radicals in human monocytes. J Agric Food Chem.

[B22] Fontaine J, Eudaimon M, Fontaine J, Eudaimon M (2000). Liquid chromatographic determination of lysine, methionine, and threonine in pure amino acids (feed grade) and premixes: collaborative study. J AOAC Int.

[B23] McLennan PL (2001). Myocardial membrane fatty acids and the antiarrhythmic actions of dietary fish oil in animal models. Lipids.

[B24] Ruiz-Meana M, Garcia-Dorado D (2003). Direct myocardial effects of fish oil on ischemia-reperfusion injury. Beyond lipid membrane composition?. Cardiovasc Res.

[B25] Herman EH, Ferrans VJ (1998). Preclinical animal models of cardiac protection from anthracycline-induced cardiotoxicity. Semin Oncol.

[B26] Kang YJ, Chen Y, Epstein PN (1996). Suppression of doxorubicin cardiotoxicity by overexpression of catalase in the heart of transgenic mice. J Biol Chem.

[B27] Undeland I, Hultin HO, Richards MP (2003). Aqueous extracts from some muscles inhibit hemoglobin-mediated oxidation of cod muscle membrane lipids. J Agric Food Chem.

[B28] Rådendal T (2004). Effect of a herring-containing diet on the antioxidant capacity of human plasma.

[B29] Nittynen L, Nurminen ML, R K, Vapaatalo H (1999). Role of arginine, taurine and homocysteine in cardiovascular diseases. Annals of Medicine.

[B30] Larsen R, Stormo SK, Dragnes BT, Elvevoll EO (2007). Losses of taurine, creatine, glycine and alanine from cod (Gadus morhua L.) fillet during processing. Journal of Food Composition and Analysis.

[B31] Sugiyama T, Kubodera M, Inoue C, Sadzuk Y (2004). Enhancing effects of unique amino acids, taurine and theanine, on the antitumor activity of doxorubicin. Proc Amer Assoc Cancer Res.

[B32] Huang X-M, Zhu W-H, Kang M-L (2003). Study on the effect of doxorubicin on expressions of genes encoding myocardial sarcoplasmic reticulum Ca2+ transport proteins and the effect of taurine on myocardial protection in rabbit. Journal of Zhejiang University SCIENCE.

[B33] Chan KM, Decker EA (1994). Endogenous skeletal muscle antioxidants. Crit Rev Food Sci Nutr.

